# Collective Defense of *Aphis nerii* and *Uroleucon hypochoeridis* (Homoptera, Aphididae) against Natural Enemies

**DOI:** 10.1371/journal.pone.0010417

**Published:** 2010-04-29

**Authors:** Manfred Hartbauer

**Affiliations:** Behavioural Ecology Group, Department of Zoology, Karl-Franzens University Graz, Graz, Austria; University of Plymouth, United Kingdom

## Abstract

The prevalent way aphids accomplish colony defense against natural enemies is a mutualistic relationship with ants or the occurrence of a specialised soldier caste typcial for eusocial aphids, or even both. Despite a group-living life style of those aphid species lacking these defense lines, communal defense against natural predators has not yet been observed there. Individuals of *Aphis nerii* (Oleander aphid) and *Uroleucon hypochoeridis*, an aphid species feeding on *Hypochoeris radicata* (hairy cat's ear), show a behavioral response to visual stimulation in the form of spinning or twitching, which is often accompanied by coordinated kicks executed with hind legs. Interestingly, this behaviour is highly synchronized among members of a colony and repetitive visual stimulation caused strong habituation. Observations of natural aphid colonies revealed that a collective twitching and kicking response (CTKR) was frequently evoked during oviposition attempts of the parasitoid wasp *Aphidius colemani* and during attacks of aphidophagous larvae. CTKR effectively interrupted oviposition attempts of this parasitoid wasp and even repelled this parasitoid from colonies after evoking consecutive CTKRs. In contrast, solitary feeding *A. nerii* individuals were not able to successfully repel this parasitoid wasp. In addition, CTKR was also evoked through gentle substrate vibrations. Laser vibrometry of the substrate revealed twitching-associated vibrations that form a train of sharp acceleration peaks in the course of a CTKR. This suggests that visual signals in combination with twitching-related substrate vibrations may play an important role in synchronising defense among members of a colony. In both aphid species collective defense in encounters with different natural enemies was executed in a stereotypical way and was similar to CTKR evoked through visual stimulation. This cooperative defense behavior provides an example of a surprising sociality that can be found in some aphid species that are not expected to be social at all.

## Introduction

Many aphid species constitute pests that inflict significant economic damage on cultivated and wild-growing plants. Their damaging influence on hosts is mainly a consequence of a very rapid reproduction that quickly leads to large aphid colonies, which are often difficult to combat by means of biological pest control. In this context, a detailed understanding of defense behavior executed by a pest and the behavior of natural enemies is of relevance for understanding both the general ecology and potential for biological pest control of aphid species. The repertoire of aphid defense against natural enemies covers a wide range of passive defense mechanisms like aposematism in combination with the ingestion or sequestering of toxins from host plants [1], [2], mechanical and physiological defenses [3], [4], avoidance reactions like walking away or dropping from host plants [5], [6], [7], [8], [9], [10], [11], [12] and active defense behavior in the form of twitching or spinning, a behavior often accompanied by forceful kicks executed with hind legs [3], [6], [13], [14], [15], [16], [17], [18], [4].

In some aphid species kicking was found to be effective in knocking parasitoid wasps away. However, the effectiveness of solitary behavioral defense depends on the enemy and on the larval stage of the defending aphid [6], [19], [20]. A commonly found protective strategy of aphids against various natural enemies is their mutualistic relationship with ants [21], [22]. Another defense strategy found in eusocial aphids comprises a soldier caste with members that are willing to risk their lives in duty of colony defense [e.g. 23], [24], [25], [26], [27], [28], [29]. There, collective defense is based on cooperating soldiers and was found to be effective in warding off or even kill attackers [30], [31], [32]. In *Pseudogrema sundanica* both, ants and soldiers defend colonies against natural enemies [31], [33]. In contrast, active defense against natural enemies at the colony level has not yet been described for group-living aphids lacking a soldier caste and a mutualistic relationship with ants.

Large aggregates like aphid colonies may be more attractive to parasitoids, kleptoparasites and different predators. This disadvantage of living in groups will be offset by the benefits associated with group living, e.g. collective defense [34], [35]. This holds true as long as the costs associated with defense reactions are low compared to the benefits gained (for example [36], [37]). Collective defense is based on cooperating members of a group, and according to Hamilton's theory of kin selection [38] should be more likely to evolve in groups consisting of members with a high relatedness. Due to the parthenogenetic reproduction of aphids whole colonies often represent clones of a single foundress. Therefore, the prerequisites for the evolution of collective defense seem to be fulfilled. This postulation recieves strong support from examples of social aphids such as the remarkable suicide behavior of infested pea aphids (*Acyrthosiphon pisum*) where dropping from host plants breaks down the reproduction cycle of parasitoid wasps [39].

Collective defense is a characteristic feature of eusocial insects [40]. However, in recent years it has become clear that this social trait is not restricted to eusocial insects like swarming ants, termites, wasps and bees, but can be found in group-living insects as well. For example, active defense by violent wriggling, biting and spitting reduces parasitoid risk in aggregations of lepidopteran larvae [41]. In some gregarious caterpillar larvae, *Hylesia sp.* and *Euphydryas phaeton*, simultaneous head jerking prevented the close approach of parasitoids [42], [43]. A similar observation was made in the gregarious sawfly, *Nediprion sertifer*, where a touch or a rapid movement of the parasitoid causes larvae to jerk synchronously [44].

In the present study collective defense was observed in natural colonies of *Aphis nerii* (Oleander aphid) and *Uroleucon hypochoeridis* (Fabricius 1779), two group-living aphid species belonging to the family of Aphididae. In encounters with natural enemies simultaneous defense occurs in the form of spinning or twitching accompanied by forceful kicks executed with hind legs. Collective defense was studied in the course of attacks of natural foliage-foraging enemies, stabbing attacks of parasitoid wasps and visual stimulation. In addition to visual signals, vibration cues may be important for the recruitment of colony members contributing to coordinated defense. For example synchronized vibration signals in the sap-feeding treehopper *Calloconophora pinguis* were found to be important in the context of predation. There, synchronized vibration signals evoke maternal defense against predators [45], [46]. Therefore, laser vibrometry was used for recording substrate vibrations generated in the course of collective defense reactions.

The aims of the present study are therefore to describe synchronized defensive behaviour in two aphid species (*Aphis nerii* and *Uroleucon hypochoeridis*) which lack both a soldier cast and a mutual relationship with ants and to determine whether collective defense is a more effective mechanism for deterring attacks than solitary defense behavior in each case.

## Results

### Solitary defense reactions

**Figure 1:**
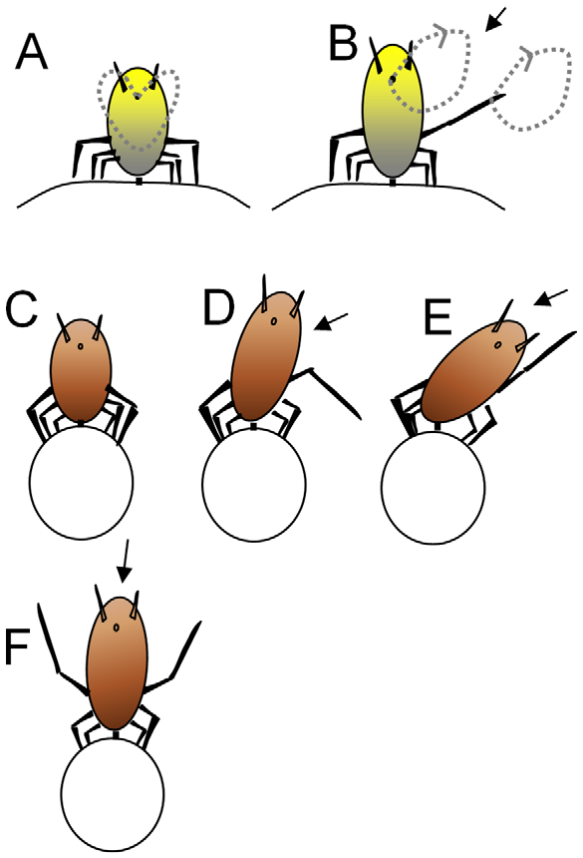
Sketch of solitary defense reactions in *A. nerii* and *U. hypochoeridis* aphids. Spinning of *A. nerii* individuals (A, B) is executed in a way so that the tip of the abdomen follows an almost circular path (dotted lines in A). **B** A visual stimulus presented from the right side (arrow in B) results in a circular movement of the tip of the abdomen and a similar circular movement of the right hind leg. **C** Resting position of *U. hypochoeridis*. **D** and **E** Successive steps sketching twitching *U. hypochoeridis* aphids when the stimulus (arrow) is presented on the right side. **F** Kicks executed by *U. hypochoeridis* when an object approaches from behind.

In both species a lifted abdomen (bucking) already constitutes the starting posture of a solitary defense reaction, which is characterized by a rapid movement of the body, termed ‘twitching’ or ‘spinning’. This behavior is often accompanied by coordinated kicks performed with one or both hind legs. Although twitching can involve powerful movements, the stylet is never removed, even when twitching is executed in close succession. In *A. nerii* the tip of the abdomen follows an almost circular path, a behavior termed spinning (Fig. 1A). During spinning the abdomen never touches the surface of the host plant. In *U. hypochoeridis* spinning was not observed, instead the whole body vigorously swings from one side to the other (twitching). In doing so the tip of the abdomen reaches out farthest. For simplicity, I further refer to this kind of solitary defense behavior observable in both aphid species by using the term ‘twitching’. Most often twitching is accompanied by forceful kicks of one or both hind legs executed towards the side of the stimulus (Fig. 1 B, D, E). In the case that a disturbing object is behind an aphid, both legs are used for forceful kicks (Fig. 1F). Twitching is driven by the front and middle pair of legs and increases the radius of action of hind legs (see dotted circles in Fig. 1B). In *U. hypochoeridis* that hind leg opposite to the stimulus often supports kicks executed with the other hind leg. Twitching in both species was never accompanied by audible sound, although sound production was described for other aphid species (see [47], [48]).

In the most cases *A. nerii* individuals twitched to the right and left side. The average duration of this behavior is only 372±50.2 ms (observed at 23°C; N = 10, visual stimulation by means of an approaching object). Because aphids are poikilothermic organisms the duration of twitching is temperature dependent and lasts 929±168 ms at an ambient temperature of 11°C (N = 14). Twitching in *U. hypochoeridis* often consists of a rapid body movement executed only towards the side the stimulus. At an ambient temperature of 25°C this behavior lasts 243±46 ms (N = 17). At a lower ambient temperature of 20°C twitching of *U. hypochoeridis* is slower and lasts 393±53 ms (N = 10). When a disturbing object is next to *U. hypochoeridis* individuals (2–3 cm) twitching is executed to both sides.

### Prerequisites for collective defense

**Figure 2:**
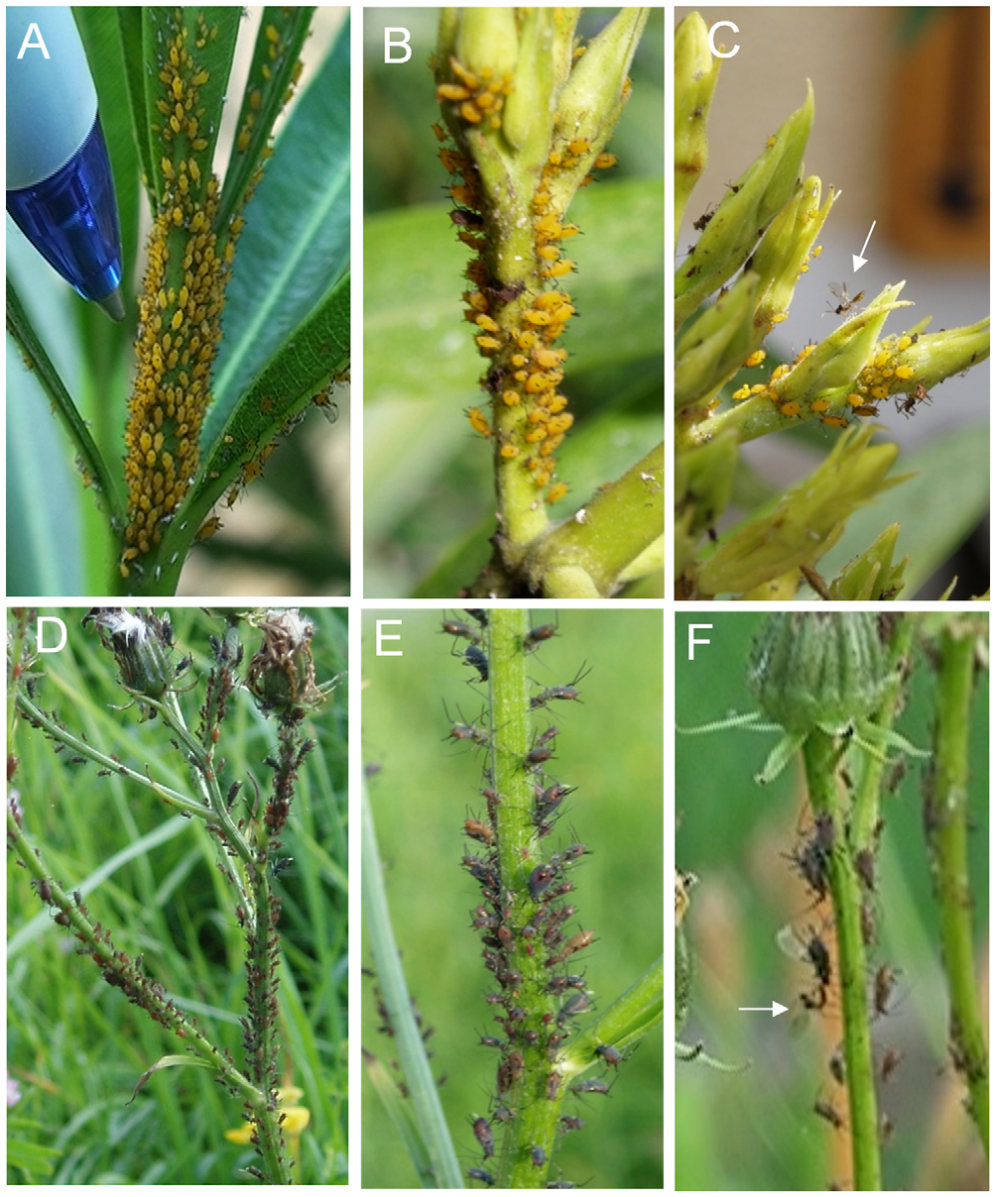
Pictures of aphid colonies and the parasitoid wasp *Aphidius colemani*. *A. nerii* colonies feeding on Oleander (**A**–**C**) and *U. hypochoeridis* colonies feeding on *Hypochoeris radicata* (**D–F**). CTKR of *A. nerii* colonies is restricted to those colonies consisting of bucking individuals (a lift abdomen) and some space left for twitching (B). The colony in A is not responding to a rapidly approaching object like a ball pen (diameter  = 12 mm, tip size  = 1 mm). **C** *Aphidius colemani* after less successful oviposition attempts. **E** Members of an *U. hypochoeridis* colony in defense posture. **F** An oviposition attempt of *Aphidius colemani* before this parasitoid was repelled from an *U. hypochoeridis* colony through consecutive CTKRs.

In the feeding position most individuals of *A. nerii* and *U. hypochoeridis* colonies preferably face downward on the stalk of their host plants (Fig. 2A, D). In both aphid species collective twitching was only found in colonies exhibiting a low to moderate aphid density (0.3±0.1 individuals per mm^2^, N = 8). In such colonies there is enough inter-individual space left for twitching. In response to a visual stimulus a collective twitching and kicking response (CTKR) could only be reliably elicited when colony members also show a lifted abdomen (bucking), a posture that is often accompanied by lifted hind legs (Fig. 2 B, E). CTKR is defined as the simultaneous response of at least two individuals of a colony and a minimum of 50% of all colony members taking part in this kind of collective response. CTKR was never observed in *A. nerii* colonies consisting of individuals sucking sap next to each other, hence very dense colonies (0.6±0.1 individuals per mm^2^, N = 8; Fig. 2A). Natural colonies often represent a mixed age distribution. Interestingly, twitching was performed by a range of instar nymphs as well as by alate and even by those females giving birth. Sometimes early instar stages were the first to respond to a stimulus. These small individuals were less demanding concerning the space necessary for twitching.

In *A. nerii* CTKR was very often observed in those colonies consisting of aphids feeding on shoots or inflorescences of Oleander (*Nerium oleander*). Leafs of Oleander were often colonized in a way so that a higher density of individuals is found close to the central vein and a lower density at the margin of the colony. Twitching in ‘leaf colonies’ was more frequently executed by those individuals located at the border of a colony. Individuals on leafs always represent different instar stages.

*U. hypochoeridis* colonies are less patchy compared to *A. nerii* and often colonies are dispersed on several terminal branches of a common host (Fig. 2D). Members of *U. hypochoeridis* colonies feed in a quite regular inter-individual distance of about 2–4 mm with enough space left for twitching (Fig. 2E). In response to visual stimulation evoked by rapidly approaching objects (tip of a chop stick) CTKR could be evoked in 94% of *U. hypochoeridis* colonies feeding on 98 branches of *Hypochoeris radicata* plants (in total 21 plants, observed at an ambient temperature of 24°C).

### Collective defense against parasitoid wasps

**Figure 3:**
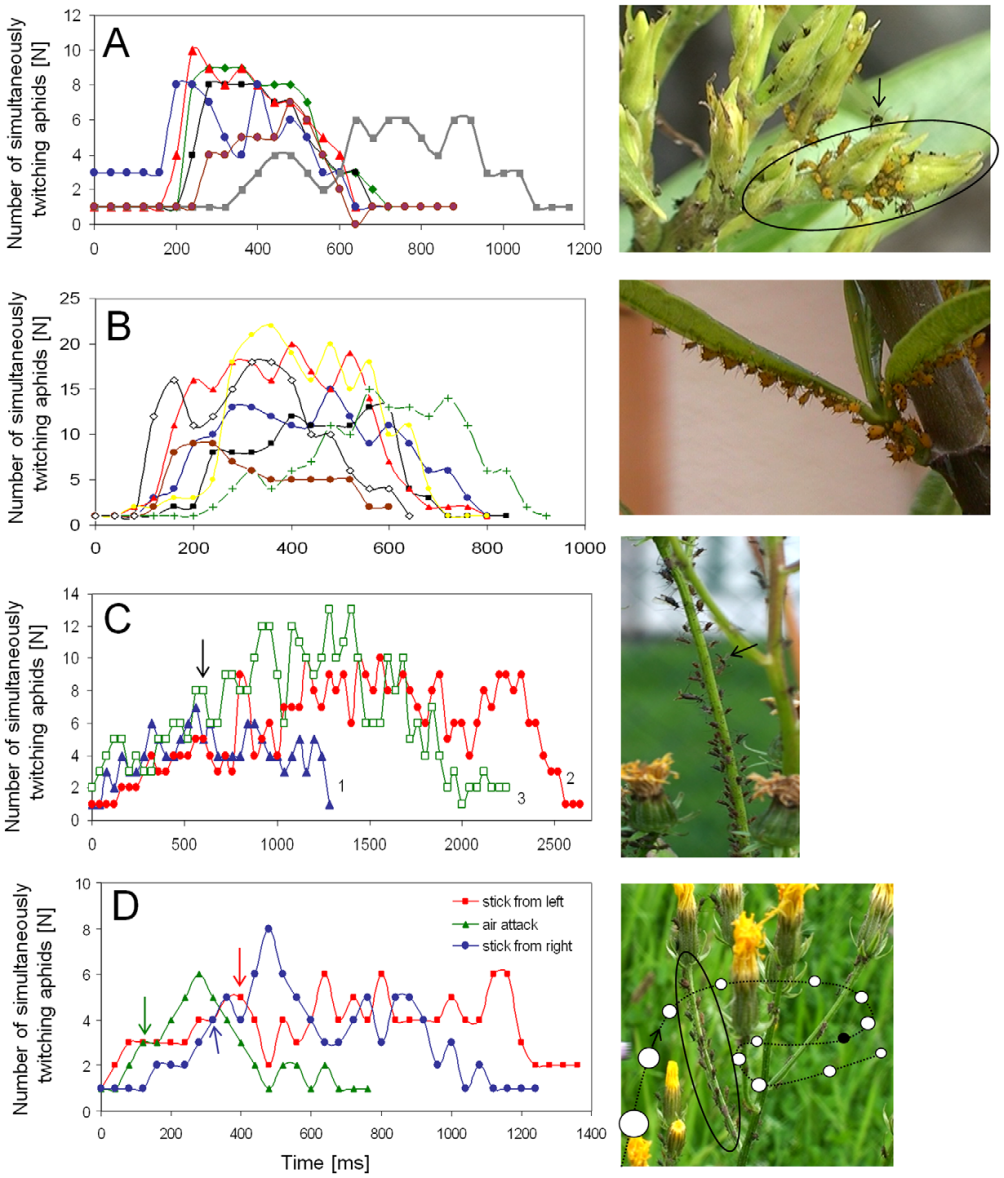
CTKR evoked by parasitoids and by visual stimulation. Time course of simultaneous twitching observed in *A. nerii* colonies (A and B) and *U. hypochoeridis* colonies (C and D). Repeated stabbing attacks of *A. colemani* evoked CTKR of the colony shown in the circle in the picture next to A. The time course of simultaneous twitching obtained from 6 stabbing attacks are shown in A. **B** Time course of CTKR of an *A. nerii* colony evoked by an approaching ball pen (see Fig. 2A). This visual stimulus was always withdrawn after the first aphids started to twitch (∼5 cm in front of the colony). A pause of about 30 sec separates successive stimulations. **C** *A. colemani* evoked CTKR of an *U. hypochoeridis* colony shown in the picture next to C. After the third CTKR this parasitoid was repelled (arrow in C). **D** CTKR of an *U. hypochoeridis* colony was evoked by a chop stick with a tip diameter of 3 mm. This stick approached this colony either from the right or from left side. CTKR of this colony was also evoked by an approaching insect (size  = 7 mm). The black dot in the flight track (dotted line) indicates the position when the first aphid started to twitch. Arrows in D indicate time points when the stick was withdrawn and the insect steered away from this colony. Data in all graphs are aligned to the first twitching individual.

The parasitoid wasp *Aphidius colemani* was never found in the centre of colonies of *A.nerii* but instead this parasitoid was found at the margins of colonies where it attacked individual aphids. When above defined conditions concerning colony density are met oviposition attempts of the parasitoid wasp *Aphidius colemani* (Fig. 2C) always evoked stereotype defense reactions like twitching and kicking in individual colony members. The probing of the host with antenna elicited solitary defense behavior of *A. nerii* individuals that evoked CTKR in the rest of the colony with a delay of about 228±85 ms (see also Fig. 3A). Sometimes it happened that CTKR was further delayed although single colony members already twitched in synchrony (see grey curve in figure 3A). After probing individual *A. nerii* colony members CTKR often spreads out in a wave-like manner. In this aphid species the generation of only one CTKR was sufficient in order to interrupt oviposition attempts of *A. colemani* and consecutive CTKRs always caused a repelling effect (see example in Fig. 2C and movie S1, observed in 5 colonies). Observations of parasitoid attacks of solitary *A. nerii* sap feeding at some distance to the next neighbour (>1.0 cm) showed that these individuals were occasionally successful in interrupting oviposition attempts through twitching and kicking. However, in the course of repeated stabbing attacks solitary individuals were unable to prevent oviposition of *A. colemani.* Interestingly, solitary aphids sometimes do not show a defense reaction at all (see movie S2) (4 individuals lacking a defense reaction; 8 individuals interrupted oviposition attempts at least one time).

Due to the small body size of *A. colemani* and a large inter-individual distance, this parasitoid wasp could stay for a while in *U. hypochoeridis* colonies without evoking collective defense reactions. However, oviposition attempts of *A. colemani* in *U. hypochoeridis* colonies evoked solitary defense reactions in those colony members surrounding the parasitoid (see first part of movie S3). In the course of local defense reactions CTKR was frequently evoked, although not always triggered by those aphids in the proximity of the wasp. Through repeated CTKR this parasitoid wasp was repelled from *U. hypochoeridis* colonies (see example in Fig. 2F, see also the second part of movie S3, observed in 6 colonies).

Nevertheless, in both aphid species this repelling effect was short lasting, because parasitoid wasps often returned to the same or a neighbor colony within tens of seconds and resumed stabbing attacks. This interplay between stabbing attacks and the repelling effect of CTKR forced *A. colemani* to repeatedly resume attacks. In *A. nerii* colonies that were dispersed on several terminal branches of Oleander repeated oviposition attempt of an *A. colemani* individual evoked 114 CTKRs within 17 min. In the presence of *A. colemani* another *A. nerii* colony generated 16 CTKR responses within 10 minutes. As a result of the repelling effect of CTKR *A. colemani* was hindered from gaining access to *A. nerii* colonies.

In such aphid-parasitoid interactions a significantly higher proportion of twitching individuals were active at the same time in *A. nerii* colonies compared to *U. hypochoeridis* colonies (mean ± SD of 8 CTKR responses: 83±14% vs. 64±3%; p<0.05; t-test). The mean duration of parasitoid-evoked CTKR is 2240±682 ms in *U. hypochoeridis* colonies and only 800±194 ms in *A. nerii* colonies. This between-species difference is highly significant (p<0.001; t-test; N = 8, ambient temperature: 22–24°C). In both aphid species individuals did not exude droplets from their cornicles after finishing CTKR. This holds true even during repetitive twitiching responses of individual colony members.

### Visually-evoked CTKR

Long time observations of single aphid colonies (20 min–1 h) showed that a spontaneous generation of CTKR is absent and CTKR is not evoked by wind that gently moves the branches of host plants. In contrast, CTKR was reliably evoked in the course of visual stimulation by use of a tip of a finger, a tip of a ball pen, or the tip of a chop stick. A rapid approach of one of these objects reliably evoked CTKR in colonies of *A. nerii* and *U. hypochoeridis* (examples are shown in Fig. 3B, D; see movies S4 and S5). Visual stimulation resulted in CTKR that rarely spreads out in a wave-like manner within a colony. Withdrawing the chop stick after aphids started to twitch resulted in a significantly higher proportion of twitching colony members in *U. hypochoeridis* colonies compared to *A. nerii* colonies (mean ± SD: 76.3±12.0% vs. 61.0±14.7%, p<0.05, Student t-test, 35 CTKRs of *A. nerii* colonies and 17 CTKRs of *U. hypochoeridis* colonies). Aphids not responding to a visual stimulus were also found outside dense regions of a colony. Interestingly, most of these inactive aphids twitched after tactile stimulation (gentle touch). Among twitching aphids, the proportion of simultaneously active individuals was significantly higher in *A. nerii* colonies compared to *U. hypochoeridis* colonies (mean ± SD: 85.1±16.6% vs. 59.1±11.7%, p<0.001, Mann Whitney U test). The duration of visually evoked CTKR was significantly shorter in *A. nerii* colonies compared to *U. hypochoeridis* colonies (mean ± SD: 782±169 ms vs. 2325±812 ms, p<0.001, Mann Whitney U test, ambient temperature: 22–25°C). A prolongation of twitching in *U. hypochoeridis* colonies is achieved by a lower degree of simultaneously twitching individuals and repeated twitching of individual colony members. Colony size in both species was positively correlated with the number of twitching aphids (*A. nerii:* cc = 0.83, p<0.001, N = 44; *U. hypochoeridis*: cc = 0.67, p<0.001, N = 27, Spearman Rank Order correlation). Interestingly, only in *U. hypochoeridis* colonies the duration of CTKR is positively correlated with the number of twitching aphids (cc = 0.706, p<0.001, Spearman Rank Order correlation, N = 27). This is in contrast to *A. nerii* colonies where the duration of CTKR was found to be independent from the number of twitching aphids (cc = −0.172, p = 0.264, N = 44, Spearman Rank Order correlation).

### Collective defense in encounters with aphidophagous larvae

**Figure 4:**
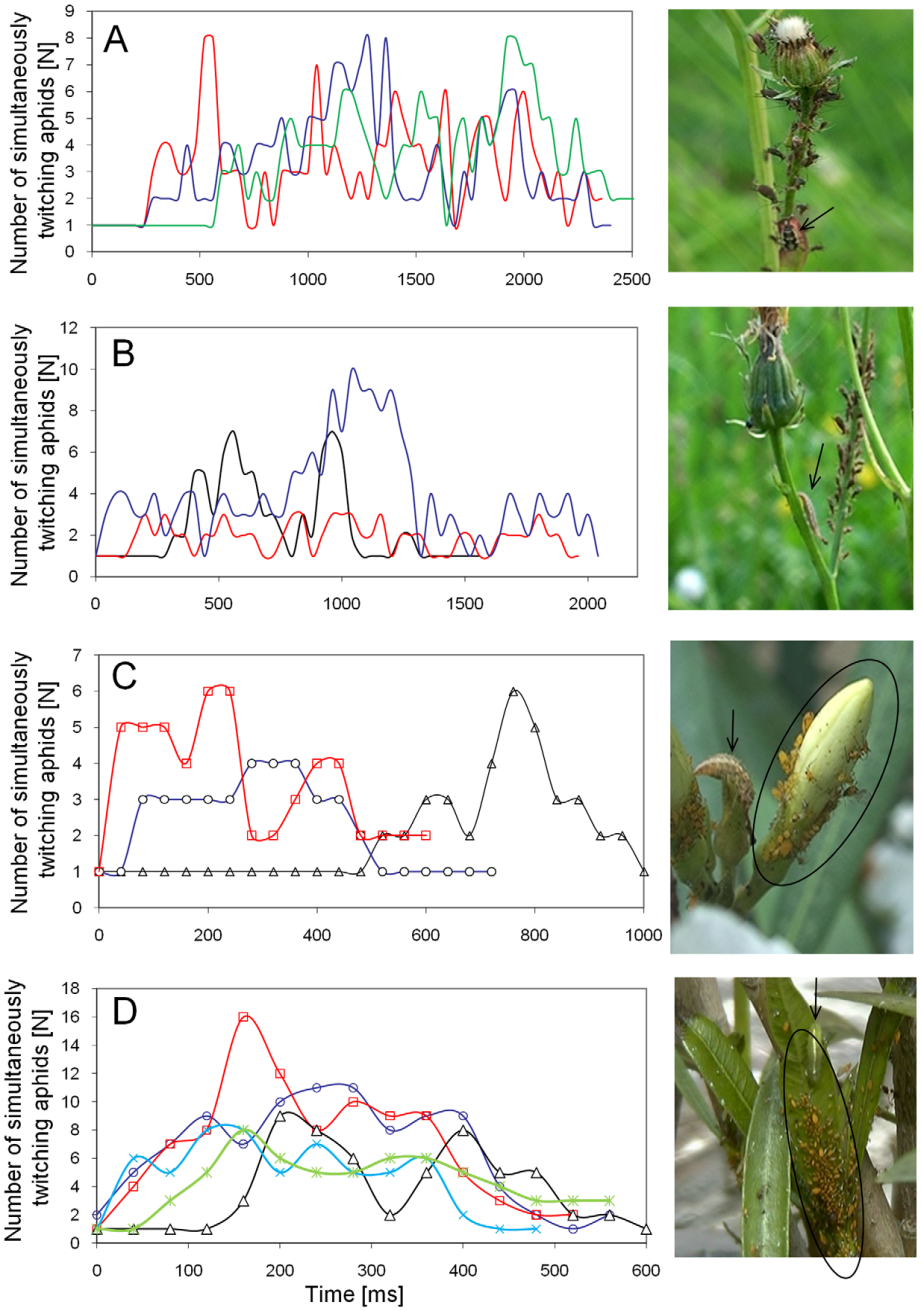
CTKR evoked by aphidophagous larvae. In *U. hypochoeridis* (A and B) colonies and in *A. nerii* (C and D) colonies CTKRs were evoked in the course of attacks of ladybird larvae (A), hemerobiid larvae (B and C) and syrphid larvae (D). Curves represent the time course of simultaneously twitching individuals belonging to those aphid colonies that are shown in the pictures next to graphs (ellipse in C and D). Arrows point to aphidophagous larvae. Curves in graphs are aligned to the first twitching individual.

CTKR was frequently observed in the course of attacks of aphidophagous coccinellid, syrphid and hemerobiid larvae (Fig. 4). In the near of aphid colonies every movement of larvae was able to evoke CTKR that lasted significantly longer in *U. hypochoeridis* colonies compared to *A. nerii* colonies (mean ± SD: *A. nerii*: 630±165 ms, N = 8, *U. hypochoeridis*: 2140±357 ms; N = 6, p<0.001; Mann-Whitney U test). In such encounters a significantly higher proportion of active colony members twitched at the same time in *A. nerii* colonies compared to *U. hypochoeridis* colonies (78±11% vs. 53±3%; p<0.001; Mann-Whitney U test). Interestingly, CTKR was often followed by a period of immobility of the attacking larvae (mean ± SD of the duration of immobility: a syrphid larvae attacking *A. nerii*: 3.9±2.1 s, N = 7; a hemerobiid larvae attacking *U. hypochoeridis*: 13.9±1.7 s, N = 5; a coccinellid larvae attacking *U. hypochoeridis*: 20.2±11.2 s, N = 5, ambient temperature: 22–25°C). In two of such predator-prey encounters the number of aphids taking part in CTKR positively correlated with the duration of larval immobility (Syrphid larvae attacking *A. nerii*: cc = 0.95, p<0.001, N = 7; hemerobiid larvae attacking *U. hypochoeridis*: cc = 0.82, p<0.05, N = 8, Spearman Rank Order correlation). Syrphid larvae foraging on leaves were often observed banging their head against substrate, a behavior that evoked CTKR in nearby aphid colonies (see movie S6).

### Habituation of CTKR

**Figure 5:**
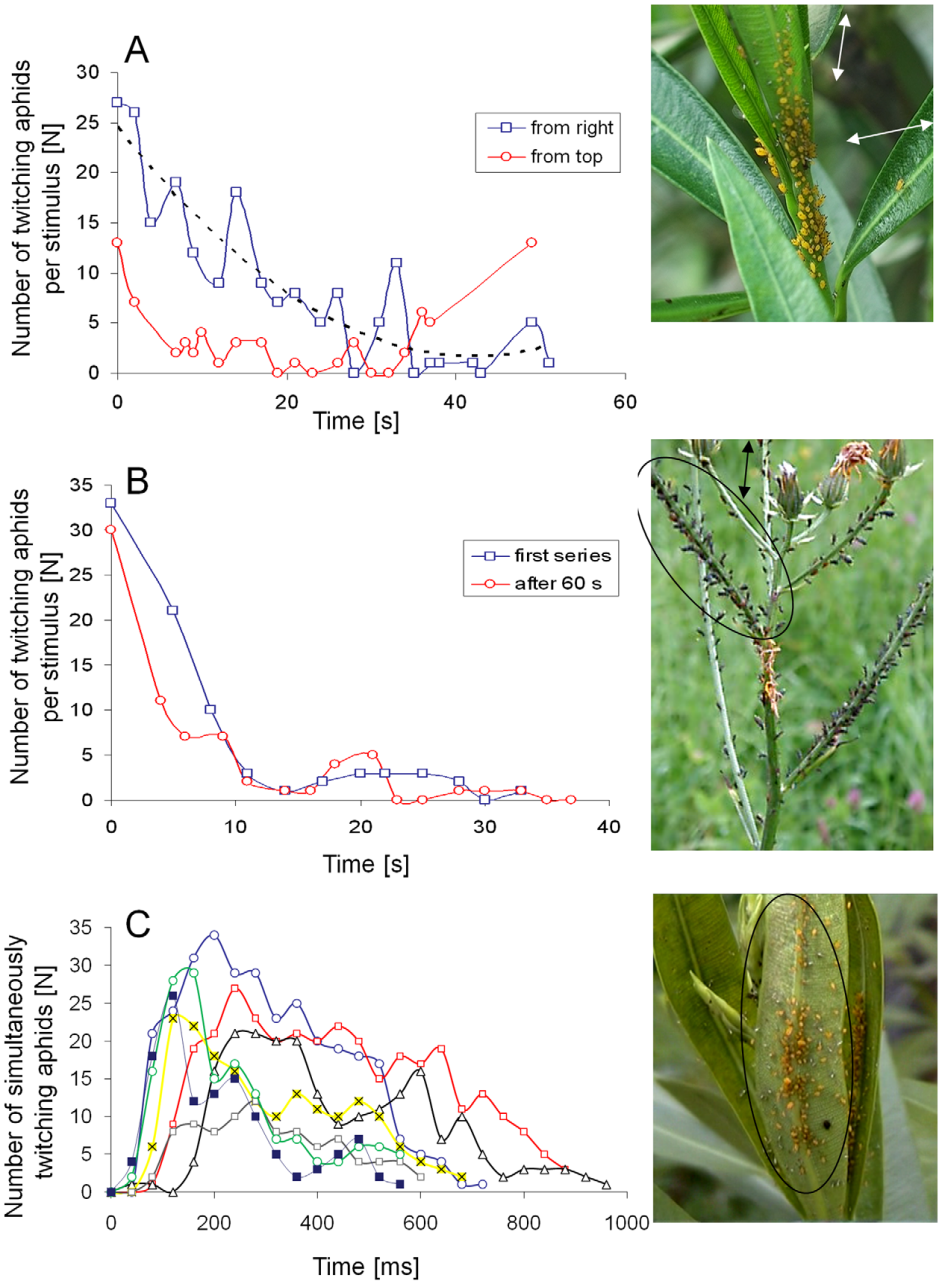
Habituation of CTKR and CTKRs evoked through substrate vibrations. Quickly moving back and forth the tip of a blue ball pen (see S1A) resulted in a habituation of CTKR of *A. nerii* (A) and *U. hypochoeridis* (B) colonies. Stimulus directions are indicated by double-arrows in the pictures next to A and B. The stimulus in A was either presented from the right side (blue curve) or from top (red curve). Introducing a pause of 15 s in *A. nerii* (last dot in the red curve in A) and a pause of 60 s in *U. hypochoeridis* restored CTKR (red curve in B). **C** Time course of CTKR evoked by substrate vibrations that were induced by gently tipping on the stalk 10 cm below the colonized leaf (ellipse in the picture next to C). Vibratory stimulations were separated by pauses of 30 s. Curves in C are aligned to the onset of the stimulus.

Visual stimulation generated by moving an object back and forth in front of *U. hypochoeridis* and *A. nerii* colonies caused a successive decrease in the number of simultaneously twitching colony members. After about 4–8 of such stimulus presentations (presented at 1 Hz in A. nerii colonies and at 0.5 Hz in *U. hypochoeridis* colonies) only a few colony members were left responding to this visual stimulus (Fig. 5 A, B). CTKR could be successfully restored by introduction of a pause of about 15 s in *A. nerii* colonies (Fig. 5A, last dot in red curve) and of about 60 s in *U. hypochoeridis* colonies (Fig. 5B). In addition, dishabituation of CTKR was reliably attained by changing the angle of the stimulation.

### Vibration-evoked CTKR

Gently tapping colonized stalks of Oleander by means of a stick induced substrate vibrations that immediately evoked CTKR in *A. nerii* colonies (see example in Fig. 5C; see movie S7). This response was observed in 7 different colonies. The earliest response to vibrations in the form of twitching aphids was observed only after ∼40 ms (one movie frame) (mean ± SD: 100±42 ms). Stronger tapping or repetitive tapping (0.5–1 Hz) resulted in a lowering of the abdomen and immobility. It was more difficult to find the right stimulus intensity to evoke CTKR in *U. hypochoeridis* compared to *A. nerii* colonies. Although a visible stimulus was absent in all vibration experiments, CTKRs were often accompanied by kicks executed with hind legs.

### Laservibrometry

**Figure 6:**
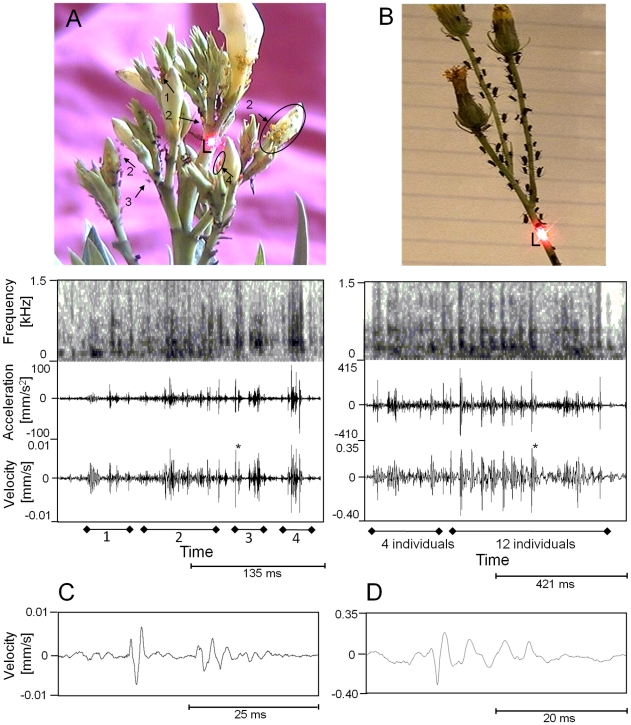
Laser vibrometry of CTKR. Lower traces in A and B represent the velocity of substrate vibrations associated with either solitary or collective twitching. Laser vibrometer focus points are labeled as L. **A** Laser vibrometry of twitching *A. nerii* individuals (arrows) and small *A. nerii* colonies (circles) feeding on an Oleander twig. Numbers below the velocity signal in A relate to labels shown in the picture above. **B** Substrate velocity generated by twitching individuals belonging to a colony of *U. hypochoeridis* dispersed on three terminal branches of a host plant (*Hypochoeris radicata*). The acceleration of the velocity signal is shown below sonograms, which were generated by use of a FFT size of 1024 points (sampling rate: 48 kHz). Twitching was evoked by visual stimulation. **C** The velocity signal marked with an asterisk in A. **D** The velocity signal marked with an asterisk in B.

The combination of laservibrometry with video observation revealed that twitching in *A. nerii* and *U. hypochoeridis* colonies generates vibratory signals that can be measured on the surface of respective host plants. Twitching-related vibrations consist of sharp velocity peaks lacking any frequency modulation (Fig. 6C, D). Twitching executed towards both sides induced vibrations that often consist of two temporarily separated velocity peaks (label 1 and 3 in figure 6A). Substrate vibrations related to twitching were measured even a few centimeters apart from twitching individuals and sometimes even on neighboring branches (Label 1 and 3 in figure 6A). Maximal acceleration amplitudes of vibration signals measured in the proximity (1–6 cm) of twitching *U. hypochoeridis* individuals of medium size are in the range of 50 to 380 mm/s^2^ (mean: 171±105 mm/s^2^, N = 11). Twitching executed by late instar *A. nerii* individuals feeding near the base of an infloresence generated maximal acceleration amplitudes in the range of 40 to 95 mm/s^2^ (mean: 58±20 mm/s^2^, N = 9, measuring spot was 0.3–1.3 cm distant from aphids).

CTKR resulted in substrate vibrations that combine to form a composite, group signal that consists of many sharp velocity peaks (Fig. 6A, B; see and listen to movie S8 and S9). Maximal acceleration amplitudes of substrate vibrations evoked in the course of CTKR were stronger compared to solitary twitching. CTKR of an *U. hypochoeridis* colony induced vibrations that were stronger at the distal part of the host plant compared to the proximal part (Maximum acceleration distal: 802±457 mm/s^2^, proximal: 407±371 mm/s^2^, N = 8 CTKRs in which 6–14 individuals took part). CTKR generated by 4–8 *A. nerii* individuals belonging to different larval stages feeding near the base of an inflorescence resulted in an average maximal acceleration amplitude of 87±43 mm/s^2^ (mean of 8 collective defense reactions).

In *U. hypochoeridis* the frequency range of vibratory signals generated in the course of CTKR was broadband with a major energy component at 102±17 Hz and a second minor peak at 311±137 Hz (N = 13). The major energy component of twitching-related vibrations in *A. nerii* was 159±11 Hz (N = 12), but the second minor peak was in a similar frequency range compared to the second minor peak of *U. hypochoeridis* (279±25 Hz, N = 12). In both aphid species some frequency components that are present in the power spectrum extent to 1600 Hz.

## Discussion

Prominent natural enemies of aphids are hoverflies [49], [50], coccinellid beetles [51], lacewings [52], cecidomyiid midges [53], spiders [54] and parasitoids [16]. Unspecific defense reactions of aphids like abdominal twitching and kicking with hind legs is common in Chaitophoridae, Pterocommatidae, Lachnidae, Callaphidae, Drepanosiphonidae and Aphidiidae [55]. Such non-lethal defensive behaviour and lower level harassment, including leg-shaking against parasitoids and predators might play an important defesive role [26]. In the current study collective defense of *U. hypochoeridis* and *A. nerii* that is based on non-lethal defensive behaviour against some natural aphid-enemies is described for the first time. In encounters with parasitoid wasps (see movies S1 and S3) and different aphidophagous larvae (see movie S6), but also following visual stimulation collective defense in the form of simultaneous twitching and kicking was frequently observed (see movie S5). In the study of Nault et al. [56] a trophobiotic relationship with ants was found to reduce defensive properties of myrmecophilous aphid species [56]. Since both aphid species investigated here are not protected by ants, results of the current study suggest that the opposite holds true as well.

There are four pieces of evidence that suggest CTKR as a means of collective defense. First, CTKR was regularly observed in the context of encounters with natural enemies. Second, the response is quick (within 100–200 ms). Third, CTKR was evoked by visual stimulation, substrate vibrations and after tactile stimulation of colony members. This multimodal excitability of CTKR may reflect an adaptation to a broad spectrum of predators and parasitoids. Fourth, habituation of CTKR prevents individuals from wasting energy in response to reappearing objects that do not represent a threat. This habituation represents a learning mechanism that keeps a colony ready for context-dependent defense reactions in a permanently changing environment, such as a meadow. The observed response decrement observable in the course of repeated stimulations is unlikely the result of fatigue because a change in the angle of the stimulus fully restored CTKR in an already habituated colony.

Both active and passive modes of collective defensive behaviour can be distinguished. Passive modes of collective defense involve dilution effects whereby the mathematical probability that any one individual will be randomly singled out by a predator decreases with group size [35], [57]. Additionally, grouping observable in *A. nerii* colonies may lower the probability of predator encounters [46]. Inactive colony members found in high-density *A. nerii* colonies may represent an example for a passive mode of collective defense. This is likely since losing a few members of well established colonies may not be as severe as losing individuals from a colony in a founder state. Members of founder colonies, on the other hand, may gain a fitness benefit by taking part in collective defense. The observed repelling effect of CTKR in parasitoid encounters may, therefore, reduce the vulnerability of certain larval stages in mixed-age colonies. The protection of offspring against predators has been proposed to be one of the most general factors selecting for group living [34], [35], [57], [58].

Colony density is not just associated with the generation of CTKR, but was also found to be an important factor for the investment in soldiers [31] and was found to reduce intraspecific defense in *Pterocomma salicis* and *Symydobius oblongus* and *Medoura viciae* [59]. In contrast, CTKR observed in low density colonies was carried out in a quite stereotypical manner resulting in similar defense reactions in encounters with different natural enemies as well as in the course of visual and mechanical stimulation. This is also reflected in the duration of CTKR and the proportion of the maximum of simultaneously twitching colony members. In both aphid species these parameters were not significantly different in encounters with *A. colemani*, aphidophagous larvae and in the course of visual stimulation (see table 1).

**Table 1:** Summary statistics of CTKR.

		*A. colemani* attacks	Aphidophagous larvae	visual stimulation
*A. nerii*	max. of simultan. twitching aphids [%]	83.4±13.7	78.4±11.7	85.1±16.6
	Duration of CT [ms]	800±194	630±165	782±169
*U. hypochoeridis*	max. of simultan. twitching aphids [%]	64.3±2.8	52.9±2.7	59.1±11.7
	Duration of CT [ms]	2240±682	2140±358	2325±812

Nevertheless, defense in both aphid species is organized in a different way. *A. nerii* makes use of chemical defense by incorporating and sequestering toxic cardenolide steroids from Oleander, which makes them unpalatable for predators [1]. Chemical weapons are also used in the genus Uroleucon. For example *Uroleucon sonchi* secretes wax from its cornicles for chemical defense against Argentine ants [60]. This kind of chemical defense was never observed in *U. hypochoeridis*, rather collective behvioural defense dominates. Since in both species an exudate was not released in the course of consecutive CTKRs, pheromones are likely not involved in collective defense.

Not only defense strategy differs between both aphid species, but also collective defense behavior in the form of CTKR reveals some significant between-species differences (summarized in table 1, p<0.05, one way ANOVA on ranks). In CTKRs generated by members of *A. nerii* colonies a higher degree of synchronized twitching compared to *U. hypochoeridis* colonies was found. In both species the number of twitching individuals responding to a visual stimulus positively correlated with colony size, but only in *U. hypochoeridis* colony size is positively correlated with the duration of CTKR. Generally, collective defense in *U. hypochoeridis* seems to be more plastic compared to *A. nerii* colonies that respond to different kinds of stimulation in a kind of all-or-nothing manner. This between-species difference likely reflects behavioral differences in how individuals in a colony respond to the twitching of other colony members and is less likely a consequence of differences of how aphid species colonize their hosts.

### The function of CTKR

In the study of Klingauf [6] defense reactions of neighbors not under direct attack of a parasitoid were described for *M. persicae* colonies. However, since their defense reactions are not directed against an immediate threat, this behavior was regarded as meaningless. In the current study repetitive CTKR was found to successfully interrupt stabbing attacks of the parasitoid wasp *A. colemani* and consecutive CTKRs were found to repel this wasp from a colony, at least for a short while. In contrast, this task is obviously impossible for single colony members (see movie S2 and S3). The observation that defense of solitary sap feeding *A. nerii* individuals were unable to prevent oviposition suggests that CTKR is increasing the effectiveness of defense on a colony level. The repelling effect of CTKR may be the outcome of a combination of visual signals that make a colony appear as a single large organism, substrate vibrations that may deter attackers and, most importantly, coordinated defense performed by those aphids in the proximity of attackers. Solitary defense reactions like twitching and kicking, when executed with sufficient force, have the potential to interrupt oviposition attempts of a parasitoid wasp that needs some time handling the host [3], [4], [6], [13], [14], [17], [18], [61]. De Farias and Hopper [61] show that defense reactions influence parasitoid handling times. A greater number of defenses per aphid attacked (*Diuraphis noxia*) was found in the parasitoid species *Aphidius asychis*, exhibiting longer handling times, compared to the parasitoid wasp *Aphidius matricariae*. However, this difference in individual defense reactions did not reduce parasitism *per se*. Therefore, further studies are required in order to demonstrate whether at all CTKR is reducing parasitism in *A. nerii* and *U. hypochoeridis* colonies compared to solitary individuals and to rule out the function of CTKR in encounters with aphidophagous larvae.

### Proximate mechanisms favoring synchronized defense

Plants are the transmission medium for vibratory signals, and are known to have complicated filtering characteristics that strongly depended on the carrier frequency of the transmitted signal [62], [63], [64], [65]. Vibratory signals characteristic for arthropods like planthoppers, leafhoppers and cydnide bugs are broad-band together with narrow-band low frequency components [66], [67], [68], [69], [70]. Twitching aphids also generate vibratory signals that are broad-band and show a major low-frequency energy component in the range of 100–160 Hz, a frequency that is similar to vibrations generated by bean and other stink bugs on respective host plants [71]. The acceleration amplitude of vibrational signals generated by twitching *U. hypochoeridis* aphids is stronger (∼10^−1^ m/s^2^) compared to *A. nerii* (∼10^−2^ m/s^2^), but weaker compared to the drumming of ants (10 m/s^2^) and stridulatory signals of cydnid bugs (0.06–8 m/s^2^). Assuming a sensitivity typical for vibratory signals of arthropods (in the range of 10^−2^ to 10^−4^ m/s^2^ [72], [73]), aphids should be able to detect twitching-related substrate vibrations of members of their own colony and very likely even those generated by neighbor colonies (located on the same host). In both aphid species CTKR was also reliably evoked by soft substrate vibrations (Fig. 5C). This result in combination with results obtained from laser vibrometry strongly suggests that vibrations generated in the course of twitching may play an important role in coordinating collective defense among members of a colony. The high degree of synchronized twitching found in *A. nerii* colonies is, therefore, likely the consequence of a quick response of colony members to visual signals and twitching-related substrate vibrations generated by others. A role of alarm pheromone in recruiting colony members cannot be completely excluded, although in contrast to the effect of tactile disturbance in *P. sundanica* [31], no exudate was released from cornicles.

The question arises of how these vibrational signals are generated. The mass of the swinging abdomen may generate a force that displaces the substrate. It is likely that for the generation of subsrate vibrations resonance properties of the host plant are exploited (for example see [70]). Since aphids do not take part in CTKR unless feeding sap, stylets may play a role in transmitting body motion into substrate vibrations.

A high synchronicity of twitching automatically leads to vibration signals that overlap in time. This group effect will facilitate the detection of twitching-related substrate vibrations. A similar enhancement of vibration signals was also found in synchronized group displays of the Neotropical treehopper *Calloconophora pinguis* (Hemiptera: Membracidae). There, vibration based communication maintains group feeding of siblings [74] and elicits maternal defense reactions [45]. Although twitching is likely to amplify synchronicity of twitching among individuals belonging to a colony, both investigated aphid species obviously respond differently to twitching-related cues. This needs to be assumed because vibration amplitudes are higher in *U. hypochoeridis*, but the degree of simultaneously twitching aphids is lower in this aphid species compared to *A. nerii*.

### Conclusions

CTKR may have evolved in successive steps beginning with kicks executed with hind legs and a raised abdomen, a behviour that shows some similarity to defecation behaviour [55]. Twitching extends the radius of kicks and increases the chance to hit a predator or parasitoid. As soon as members of a colony respond to defense behaviour executed by other colony members with twitching and kicking, synchronized defense behaviour in the form of CTKR is the consequence. An enhanced repelling effect in parasitoid encounters will result in inclusive fitness benefits gained by those individuals belonging to a colony. This selective force promotes colony formation and probably led to the collective defense behaviour that can be found in *A. nerii* and *U. hypochoeridis*. This, however, reveals a surprising sociality that is based on cooperating individuals, which, according to Hamilton's kin selection theory, is more likely to evolve when co-operators exhibit a high relatedness and the benefits of cooperation are higher than the costs associated with cooperation [38]. Although repeated forceful twitching can be energetically demanding, the costs associated with escape responses will be much higher [16], [39], [75]. Since the genetic relatedness between colony members of aphid species investigated in the current study is unclear, it remains speculative whether inactive colony members are the offspring of a different foundress. Nevertheless, a strong selective pressure, exerted by natural enemies, appears to promote the retention of selfish interests within a group when the group as a whole is more successful in colony defense.

## Materials and Methods

### Animals

*Aphis nerii* sap-feeds on Oleander (*Nerium oleander*) and milkweed plants and has an aposematotic yellow body [1], black legs and a pair of black siphunculi (cornicles) (Fig. 1A, B). Adults of this aphid species are 1.5–2 mm in size. Adults of *Uroleucon hypochoeridis* are bigger (3–4 mm) with a reddish body color and black siphunculi. This aphid species feeds on *Hypochoeris radicata* (also known as hairy cat's ear, gosmore) (Fig. 1C, D), a herbaceous plant that is abundant in many meadows. These aphids preferably colonize terminal parts of the plant and inflorescences. Females of both aphid species are viviparous. Ants were never seen on Oleander, in contrast to *Hypochoeris radicata*, where ants belonging to different species regularly evoked brief solitary defense reactions after contact with *U. hypochoeridis* individuals.

Initially, species identification of parasitoid wasps was quite difficult in the field. Therefore, encounters of aphids with different wasps as well as with aphidophagous larvae were first filmed and afterwards some exemplars of attacking organisms were caught and submersed in alcohol. These specimens were identified as *Aphidius colemani* (Braconidae, subf. Aphidiinae), a solitary oligophagous aphid parasitoid [76], [77] preferably infesting first- and second instar nymphs of *Myzus persicae* (Sulzer), and *Aphis gossypii* (Glover) (Hemiptera: Aphididae) [78]. This parasitoid allows his host to further develop before eating it from inside and leaving a ‘mummy’ behind (koinobiont) and was frequently found on Oleander as well as on *H. radicata*. In addition, different larval stages of aphidophagous syrphid (hover fly), chrysopid (green lacewing), hemerobiid (brown lacewings) and coccinellid larvae (ladybird larvae) were found on both host plants.

### Behavior studies

With the exception of laser vibrometry all results are obtained from field observations carried out in a natural habitat. Therefore, observations are based on natural situations neither manipulating aphid colonies nor the abundance of natural enemies. *A. nerii* was observed feeding on white flowering Oleander cultured in barrels. During winter times Oleander plants were incubated in a dry cellar. As soon as night temperatures raised above 10°C Oleander was cultured outdoors. *U. hypochoeridis* colonies were observed on two separate grass meadows each about 2000 m^2^ in size and about 100 m apart from each other. All observations were made from June to August 2009 in Ligist, a village located in the province of Styria (Austria). Aphids and their host plants were never treated with any pesticides or other chemicals.

In order to study defense behavior in encounters with natural enemies movies of collective defense reactions were made using a camcorder (HDR-HC7E, Sony Inc.) mounted on a tripod. Collective defense was also visually evoked by means of an approaching black-colored tip of a chop stick (tip diameter  = 2.5 mm; total length  = 26 cm) or an approaching ball pen. Video material showing interesting scenes (8 hours in total) was analysed in a frame per frame manner (25 fps) using Virtual dub V.1.92 (www.virtualdub.org).

Twitching of aphids is very rapid and often accompanied by fast leg movements (kicks). In order to properly study solitary defense behavior, additional video observations were made at lower ambient temperatures (11°C). The density of *A. nerii* colonies was determined by means of ImageJ after spatial calibration of images (http://rsb.info.nih.gov/iJ).

### Laservibrometry

A portable laservibrometer (PDV-100, Polytec Inc. Germany) in combination with a video camera (HDR-HC7E, Sony Inc., 25 fps) was used for the measurement of substrate vibrations produced by twitching individuals. The output of the laservibrometer was digitized with a sampling rate of 40 kHz using an AD converter (Powerlab, AD-Instruments Inc. Germany) connected to a laptop (Maxdata Pro, Germany). The velocity of substrate vibrations was recorded on terminal branches of respective host plants. The first order derivative of the velocity signal yields the acceleration of substrate vibrations. Video recordings were synchronized with vibrometer signals by logging the exact video time in the recording software (Chart 5.0 AD-Instruments Inc). In order to increase the quality of laservibrometer signals, a reflecting tape was mounted on different measuring spots on the stalks of colonized host plants. The spectral content of vibration signals was analyzed by calculating the power spectral density after FFT transformation (calculated in Chart 5.0, AD-Instruments Inc. Germany, using a window size of 4069 points). All laservibrometer measurements were carried out in the lab using colonized terminal branches of host plants that were cut one or two days before measurement (∼40 cm in length). In order to keep aphids and their host plants as vital as possible, all stalks were placed in a florist's water tube, which was exposed to natural weather conditions. Laser vibrometry was performed in a quiet room on a vibration damped desk (ambient temperature: 24°C). Defense reactions of aphids were evoked by visual stimulation generated by moving objects (the tip of a chop stick or a ball pen).

### Statistics

Analyses were conducted in Sigma Plot (V. 11, Systat software inc.). Before application of a statistical test data distribution was checked for normality by use of a Shapiro-Wilkinson test. If normality was absent non-parametric tests were performed.

## Supporting Information

Movie S1 Stabbing attacks of the parasitoid wasp *A. colemani* evokes CTKR of an *A. nerii* colony. CTKR is also evoked by wing beats (second 22 in the movie).
(4.45 MB MOV)

Movie S2 *A. colemani* attacks a solitary feeding *A. nerii* individual.
(5.37 MB MOV)

Movie S3 The parasitoid wasp *A. colemani* evokes solitary defense reactions in *U. hypochoeridis*. In the second part of this movie (starting after 16 s) the same colony wards off *A. colemani* through CTKR.
(6.93 MB MOV)

Movie S4 Visual stimulation evoked CTKR of an *A. nerii* colony. Note that wind caused some movement of the host plant.
(0.98 MB MOV)

Movie S5 Visual stimulation evoked CTKR of an *U. hypochoeridis* colony.
(1.79 MB MOV)

Movie S6 A syrphid larvae evokes CTKRs of an *A. nerii* colony.
(3.02 MB MOV)

Movie S7 Substrate vibrations evoked CTKRs of an *A. nerii* colony.
(0.73 MB MOV)

Movie S8 Laser vibrometry of CTKR of an *U. hypochoeridis* colony feeding on the terminal branches of *Hypochoeris radicata*. The signal of the laservibrometer is on the audio track of this movie.
(0.96 MB MOV)

Movie S9 Laser vibrometry of twitching *A. nerii* individuals as well CTKR of colonies feeding on terminal branches of Oleander. The signal of the laservibrometer is on the audio track of this movie.
(0.91 MB MOV)
